# Immersive virtual reality for reducing public speaking anxiety in students accessing a university psychological counseling service: protocol for a randomized controlled trial

**DOI:** 10.1186/s40359-026-04418-4

**Published:** 2026-03-23

**Authors:** Gianluca Mariano Colella, Ellen Silvestro, Vincenzo Cosentino, Vincenzo Capparelli, Antonio Gravina, Loris Barbieri, Angela Costabile, Fabio Bruno, Francesco Craig

**Affiliations:** 1 Department of Cultures, Education and Society, University of Calabria, Rende, Italy; 2 University Psychological Counselling Service (UPCS), University of Calabria, Rende, Italy; 3 Department of Mechanical Energy and Management Engineering (DIMEG), University of Calabria, Rende, Italy; 4 Department of Physics, University of Calabria, Rende, Italy

**Keywords:** Public Speaking Anxiety, Clinical Trial, Virtual Reality, Psychological Counseling, User studies

## Abstract

**Background:**

Public Speaking Anxiety (PSA) is a form of social anxiety spread among university students, negatively affecting both academic performance and overall psychological well-being. Virtual Reality (VR)–based interventions combined with Cognitive Behavioral Therapy (CBT) represent a promising approach for treating PSA, as they enable gradual and controlled exposure to feared social situations by simulating realistic audiences and environments. The primary aim of this study is to evaluate whether the use of VR as a support to standard psychotherapy is effective in managing anxiety and PSA in university students who access the University Psychological Counseling Service (UPCS).

**Methods:**

This article outlines the study protocol for a randomized controlled trial (RCT). Participants will be divided into two groups: (1) a control group receiving psychological counseling, and (2) an experimental group that receives psychological counseling integrated with VR interventions based on 360° video scenarios. The intervention will include two VR-based modules: a VR–Exposure and Response Prevention (ERP) module, designed to provide graded immersive exposure to anxiety-provoking scenarios, and a VR–Acceptance and Commitment Training (ACT) module, aimed at enhancing mindfulness and psychological flexibility through guided experiential practice. Psychological and physiological data collected during the sessions will be analyzed to compare the effectiveness of VR-integrated psychotherapy with traditional counseling alone.

**Discussion:**

This study will introduce a novel VR-based ERP and ACT training protocol designed to reduce public speaking anxiety in university students by integrating innovative, immersive VR scenarios within a psychological counseling context. The proposed approach may be particularly relevant for university settings, as it offers a scalable, standardized, and controllable VR-supported counseling protocol aimed at reducing PSA, enhancing students’ academic functioning (e.g., oral exams and presentations), and strengthening the overall effectiveness of university psychological counseling services.

**Trial registration:**

This protocol was registered at the Clinical Trials.gov (Identifier: NCT07351409) on January 16, 2026.

**Supplementary Information:**

The online version contains supplementary material available at 10.1186/s40359-026-04418-4.

## Introduction

In recent years, the rising prevalence of mental health difficulties among university students has emerged as a critical public health concern, prompting growing attention from researchers, higher-education institutions, and mental health services [1]. Recent epidemiological studies have shown that university students often report high levels of stress, generalized anxiety [2], and social anxiety [3]. Social anxiety disorder, particularly Public Speaking Anxiety (PSA), is highly prevalent among students worldwide, affecting over 60% in surveys across the USA, Brazil, and Finland [4]. This fear often emerges in adolescence, causing significant distress and physical symptoms, highlighting the need for interventions to reduce speaking-related anxiety, referred to as Public Speaking Anxiety (PSA). Multiple factors may contribute to increased anxiety among university students, including academic pressure, fear of failing exams, interpersonal difficulties, and the perceived need to succeed after graduation [5]. Providing students with effective support for managing their concerns and anxiety symptoms can promote both their personal well-being and their academic success. In this context, University Psychological Counseling Service (UPCS) gained a central role in responding to the growing demand for psychological and/or psychotherapeutic support, particularly during the pandemic and post-pandemic periods [6–8].

Among the psychotherapeutic approaches for anxiety, Cognitive Behavioral Therapy (CBT) has demonstrated robust empirical support and clinical efficacy [9]. First and second waves of CBT employ techniques such as systematic desensitization, applied relaxation, targeting the somatic symptoms of anxiety, as well as skills training and cognitive restructuring aimed at modifying dysfunctional thought patterns. The third-wave CBT models, such as Acceptance and Commitment Therapy (ACT), have expanded the therapeutic focus beyond symptom reduction to encompass core processes of psychological flexibility, by integrating mindfulness and acceptance principles to foster adaptation and resilience in the face of distressing experiences [2].

ACT is a process-based behavioral intervention that aims to enhance psychological flexibility, defined as the capacity to remain in conscious contact with the present moment while engaging in effective, values-guided action [10, 11]. The core processes underpinning ACT are acceptance, cognitive defusion, present-moment awareness, self-as-context, values clarification, and committed action. Within the context of public-speaking anxiety, ACT supports individuals in reducing defensive avoidance and in approaching performance-related cues with openness and behavioral willingness, thereby enabling more adaptive engagement with anxiety-eliciting situations [12].

Conducting exposure exercises for PSA during psychotherapy sessions has historically been challenging or even impractical, as it requires access to and control over a live audience [13]. Immersive Virtual Reality (IVR) technology can overcome this limitation by enabling safe, controllable, and repeatable virtual environments, in which social-evaluative stimuli, such as the presence of an audience in a classroom, can be systematically manipulated. These technologies can facilitate emotional regulation, behavioral change, and the integration of lived experiences by providing immersive, embodied, and repeatable simulations, thereby activating both cognitive and affective processing systems and fostering learning and desensitization in a realistic yet secure context [14, 15]. These possibilities allow us to overcome real setting limitations and lead to the development of Virtual Reality Exposure Therapy (VRET) for the treatment of PSA. Through VR-based exposure, the feared stimulus can be precisely controlled and gradually modulated. A growing body of empirical studies [16–19] demonstrated that VR-based exposure is associated with significant reductions in PSA symptoms, yielding clinical benefits often maintained over time. More recently, research has begun integrating VR with ACT-based practices. A randomized controlled trial [20] showed that a brief VR-based ACT intervention combining graded virtual exposure with ACT-consistent guidance (e.g., mindful noticing, acceptance, and cognitive defusion) resulted in a significant reduction in social and public speaking anxiety and increases in psychological flexibility among university students. These findings highlight the potential of VR not only as a vehicle for exposure but also as an effective platform for delivering ACT-consistent mechanisms of change in an immersive and experiential format.

Despite these advances, the literature on VR-based interventions for PSA and social anxiety remains limited by several methodological shortcomings. Studies rely primarily on self-report instruments, which mainly capture the subjective dimension of anxiety and provide limited insight into underlying physiological or behavioral processes [9, 13]. Moreover, VR-based interventions are rarely compared directly with established therapist-delivered interventions, making it difficult to determine whether the VR-based element offers added therapeutic value beyond traditional cognitive-behavioral approaches [4, 16]. A further limitation concerns the scarcity of long-term follow-up assessments, which restricts conclusions regarding the durability of treatment gains.

To extend the current knowledge and to test the efficacy of application-based VR interventions, the present experimental protocol is designed to overcome some of the methodological limitations previously identified in the literature. Within this framework, UPCS provides an optimal first-line intervention setting for implementing VR, offering access to a clinical environment in which immersive scenarios can be used to simulate feared social and public-speaking situations. This study is expected to contribute to addressing the current lack of evidence on CBT combined with 360° video–based VR exposure for PSA in university students and to provide preliminary support for a novel VR-based ERP and ACT protocol specifically tailored to a university counseling context.

## Method

### Aim

We adopt a two-arm randomized controlled trial (RCT) design to investigate whether VR-delivered controlled exposure can complement standard psychological counseling in addressing PSA and general anxiety symptoms among students accessing the UPCS. The findings are expected to provide evidence regarding the effectiveness of integrating VR-based exposure as an adjunct to Exposure and Response Prevention–Acceptance and Commitment Therapy (ERP-ACT) within counseling to manage anxiety and stress in university students.

It is hypothesized that:

- Primary Outcome - Students receiving brief psychological counseling supported by ERP and ACT training combined with VR-based exposure exhibit significantly greater reductions in PSA and anxiety levels from baseline (T0) to post-intervention (T1), compared with students receiving standard psychological counseling alone.
- Secondary Outcome - The two VR components (ERP and ACT) are expected to exert differential effects. Specifically, the relaxation modules should primarily reduce physical tension and physiological arousal, whereas the immersive exposure modules should mainly target the psychological aspects of public speaking, leading to lower fear and reduced perceived mental effort during performance-related scenarios. Physiological data are continuously recorded during each intervention session to assess patterns of arousal and recovery and to examine whether VR-supported counseling is associated with different within-session responses compared with counseling alone.
- Other Pre-specified Outcome - These benefits are expected to be maintained over time; therefore, students in the VR-ERP and VR-ACT training conditions are expected to continue to exhibit better outcomes at the 3-months follow-up assessment (T2), compared with students receiving standard counseling alone.

### Design and setting of the study

The study is a two-arm randomized controlled trial with parallel, independent experimental and control groups. Both groups undergo a brief standard counseling program consisting of five individual sessions delivered by trained clinicians within the UPCS. The experimental group receives the same brief standard counseling intervention, complemented by two immersive VR modules delivered through 360° video-based scenarios: (a) a VR–Exposure and Response Prevention (VR-ERP) component providing graded and controlled exposure to anxiety-provoking public-speaking situations, and (b) a VR-Acceptance and Commitment Training (VR-ACT) component offering an ACT-based mindfulness training aimed at down-regulating physiological arousal and enhancing adaptive coping. The control group receives the standard counseling protocol only.

Psychological assessments are conducted at baseline (T0), post-treatment (T1), and at follow-up (T2), allowing evaluation of change over time and between-group differences. Physiological parameters are continuously recorded during each session to capture objective indices of arousal and recovery alongside self-report outcomes. This design enabled testing whether adding immersive, VR experiences to routine counseling provides benefits beyond standard care alone.

### Sample size

A priori sample-size estimation is conducted to determine the number of participants required to detect the expected effects of the VR-based exposure intervention on the primary outcome measures. Evidence from a systematic review [21], which examined virtual reality–based interventions targeting public-speaking anxiety in student populations, indicates that VR exposure procedures typically yield moderate-to-large reductions in anxiety symptoms compared with control or baseline conditions. Reported effects across the studies reviewed are consistent with the magnitude commonly observed in the broader VRET literature, supporting the assumption of a moderately large effect size for the planned between-group comparison.

Based on this evidence, we adopt an expected effect size of Cohen’s d = 0.75 for the between-group differences in change scores from baseline to post-intervention between the VR exposure and control groups. A power analysis conducted using G*Power 3.1 (two-tailed independent-measures t-test, α = 0.05, power = 0.80) indicated that 29 participants per group would be required to detect an effect of this magnitude.

To ensure adequate statistical power after accounting for participant attrition, commonly estimated at 15%-20% in VR-based intervention studies, we plan for an upper-bound dropout rate of 20%. Accordingly, the required number of participants is adjusted to 36 participants per group, resulting in a total target sample of 72 participants [22]. This sample size is expected to provide sufficient statistical sensitivity to detect clinically meaningful effects of the VR intervention while remaining feasible for a single-site randomized controlled trial.

### Participants and recruitment

Potential participants are identified among university students who contact the UPCS. Each student undergoes a structured screening interview conducted by a research psychologist. During this interview, the aims and procedures of the study are explained, questions are addressed, and the student’s eligibility is assessed. The screening serves to rule out conditions requiring immediate or specialized care; in such cases, students are referred to the appropriate clinical services within or outside the university, in accordance with standard UPCS referral pathways.

Eligible participants are university students aged 18 years or older who seek counseling at the UPCS and report clinically relevant anxiety in performance-related contexts, particularly public speaking.

Inclusion criteria are: (a) current enrolment at the university; (b) request for psychological counseling at the UPCS; (c) age ≥ 18 years; (d) fluency in Italian; (e) provision of written informed consent; and (f) at least moderate levels of anxiety and perceived stress as indicated by standardized measures, specifically, Personal Report of Public Speaking Anxiety (PRPSA) scores between 85 and 110 and State-Trait Anxiety Inventory (STAI) scores between 35 and 50.

Exclusion criteria are: (a) current psychotic disorder; (b) acute suicidal ideation or behavior; (c) severe substance dependence; (d) severe sensory or motor impairments that could interfere with the safe use of VR equipment; (e) inability to attend the planned sessions; and (f) concurrent participation in other specialized psychotherapy programs.

After completion of the screening interview, participants are randomized (1:1) to the experimental (counseling + VR) or control (counseling only) group using a computer-generated randomization sequence. Allocation is concealed using sequentially numbered, opaque, sealed envelopes or a password-protected electronic system, which is accessed only after participant enrollment and completion of baseline (T0) measures.

Participants are considered withdrawn from the study if any of the following occurs: (1) voluntary withdrawal at any time without the need to provide a reason; (2) emergence of adverse effects or discomfort judged to make continuation inappropriate; (3) detection of a major protocol deviation; or (4) any circumstance that, in the investigators’ clinical judgment, could compromise the participant’s health safety, or well-being. The date and reason for withdrawal will be systematically recorded in a paper-based case report form (CRF).

Recruitment is anticipated to be completed by December 2026.

### Ethics and privacy

Written informed consent will be obtained from participants prior to study participation, following a comprehensive explanation of the study aims, procedures, and the provision of written information. The study received ethical approval (No. 112518/2025) from the Institutional Review Board Committee (IRBC) of the University of Calabria and is registered with the ClinicalTrials.gov (NCT07351409) on January 16, 2026. Data will be collected through a secure, encrypted digital platform and stored on institutional servers in compliance with General Data Protection Regulation (GDPR) and institutional ethics guidelines. All datasets used for analysis will be fully de-identified using unique participant identification codes.

### Assessment instruments

Participants complete a self-administered questionnaire to collect sociodemographic and academic data, including gender, age, degree programme, and disciplinary area (e.g., STEM, humanities, social sciences), year of study, and enrollment status (in-course/out-of-course).

#### Psychological assessment

The protocol involves the assessment of participants through the administration of standardized self-report psychological tests designed to evaluate levels of public speaking anxiety and state and trait anxiety levels.

The instruments are:

- The Italian version of the *Personal Report of Public Speaking Anxiety* (PRPSA) [23] is a self-report questionnaire designed to measure the level of anxiety individuals experience when speaking in public. The scale assesses multiple dimensions of public speaking anxiety, including cognitive, physiological, and behavioral components, capturing both anticipatory anxiety and anxiety during performance. The PRPSA consists of 34 items, each rated on a 5-point Likert scale (1 = strongly disagree to 5 = strongly agree). Items reflect feelings of tension, nervousness, and physiological arousal (e.g., “My hands tremble when I try to handle objects on the podium”), as well as negative self-appraisal and fear of audience evaluation. Total scores range from 34 to 170, with higher scores indicating greater levels of public speaking anxiety. Conventionally, scores above 131 suggest high anxiety, scores between 98 and 131 indicate moderate anxiety, and scores below 98 indicate low anxiety. The Italian version maintains strong psychometric properties, with high internal consistency (Cronbach’s α > 0.90) and good test–retest reliability. It has been validated for use among university students and adults, proving useful in both clinical and research contexts, examining performance-related social anxiety and communication apprehension.
- The Italian version of the *State-Trait Anxiety Inventory*,* Form Y* (STAI) [24, 25] was adopted to measure two distinct dimensions of anxiety: (i) State Anxiety (S-Anxiety), a temporary emotional condition characterized by feelings of tension, apprehension, and heightened autonomic nervous system activity, reflecting how the respondent feels at the present moment; and (ii) Trait Anxiety (T-Anxiety), a relatively stable disposition to perceive situations as threatening and to respond with anxiety across time and contexts. Each subscale consists of 20 items, rated on a 4-point Likert scale (ranging from 1 = almost never to 4 = almost always for trait anxiety, and 1 = not at all to 4 = very much so for state anxiety). Scores range from 20 to 80 for each subscale, with higher scores indicating greater anxiety levels. The Italian adaptation has demonstrated good psychometric properties, including high internal consistency (Cronbach’s α typically between 0.86 and 0.95) and construct validity consistent with the original instrument, making it one of the most widely used anxiety measures in both clinical and research settings in Italy.

The psychological tests used to assess the students will be administered at baseline (T0), post-treatment (T1, at the end of the 4-week intervention period), and after 3 months (T2, follow-up).

#### Physiological assessment

The EmbracePlus wristband is a research-grade, wrist-worn wearable device designed to continuously monitor a range of physiological parameters, providing real-time access to raw sensor data suited for scientific, clinical, and real-world studies. Its built-in sensors include photoplethysmography (PPG) for blood volume pulse (BVP), from which heart rate (HR), inter-beat interval (IBI), and heart rate variability (HRV) are derived, electrodermal activity (EDA or skin conductance) as an index of autonomic arousal, a 3-axis accelerometer to track motion and activity, and a peripheral skin-temperature sensor. The device samples EDA at 4 Hz and PPG at 64 Hz, and it has demonstrated high correlations with clinical-grade equipment for HR and HRV under controlled conditions (though less so for EDA under movement) [26]. Thanks to its wrist-worn design, on-board data storage, and Bluetooth syncing capabilities, the EmbracePlus enables extended ambulatory monitoring of physiological responses in naturalistic settings, enabling studies of stress, emotion, cognitive load, and behavioral states in real time. Data were collected during four intervention sessions, from Session 1 (T0; baseline) to Session 4 (T1), corresponding to the end of the 4-week intervention period.

### VR materials

A custom VR application was developed using Unity (version 2023) and deployed on the Meta Quest 3 head-mounted display (HMD). The Quest 3 is a fully standalone and wireless HMD, allowing participants to move freely without being physically tethered to an external computer. The system provides full visual isolation from the real-world environment and delivers a highly immersive first-person experience.

Participants are placed in a realistically reconstructed academic setting, which includes either a virtual classroom or a lecture theatre, where they are positioned in front of a virtual professor or an examination committee and an audience of virtual observers. Participants are required to engage in a structured oral examination or public speaking task. The primary goal of the system is to induce and study public speaking anxiety under controlled, ecologically valid experimental conditions.

The virtual professor and audience are animated and temporally synchronized with audio output to enhance perceived realism and social presence. The system ensures consistent experimental conditions across participants while allowing controlled variation of environmental and social parameters.

For safety and ethical compliance, participants can immediately terminate the VR session at any time by simply removing the headset, which instantly ends the immersive experience. To ensure high experimental control and to prevent simulator-induced artifacts that could confound psychological and physiological measurements, the VR application was optimized to maintain a stable high refresh rate under all experimental conditions. In particular, the system was designed to support parametrically variable audience sizes without performance degradation, as audience size represents a key experimental stressor [27]. To meet these constraints, the rendering pipeline was implemented using GPU instancing for efficient large-scale replication of audience avatars and GPU-based animation techniques to offload character animation from the CPU [28]. This architecture enables real-time scalability of animated virtual agents while preserving consistent frame timing and minimizing motion-to-photon latency. These optimizations ensure that observed variations in biometric and physiological responses can be attributed to experimental manipulations rather than to technical artifacts such as frame drops, stuttering, or rendering instability.

A dedicated PC-based control software application, developed using Microsoft Visual Studio, allows the experimenter to monitor, manage, and customize the VR session in real time. Through this interface, the experimenter can configure multiple key experimental parameters, including:

- the type of virtual environment (e.g., classroom or lecture theatre);
- the number of virtual audience members;
- the general structure and difficulty of the speaking or examination task.

The control software communicates bidirectionally in real time with the VR application, enabling the dynamic delivery and modulation of stimuli, questions, and scenario changes during the session. All experimental parameters are automatically time-stamped and logged for subsequent analysis. The control software integrates with OpenAI’s language model through an Application Programming Interface (API), enabling the dynamic and personalized generation of examination questions. The experimenter can enter high-level natural language prompts describing the participant’s academic background (e.g., *“Ask the student about a second-year engineering class”*). The language model then generates a contextually appropriate academic question, which is transmitted in real time to the VR application and delivered verbally by the virtual professor to the participant. This approach enables:

- high variability and adaptability of task content;
- personalization based on participant characteristics and academic context;
- increased ecological validity of the simulated social interaction.

All input prompts and corresponding AI-generated examination questions are automatically stored and time-stamped in the system log for later quantitative and qualitative analyses.

During each VR session, participants wear a medical-grade wrist-worn biometric device (EmbracePlus), part of the Empatica Health Monitoring Platform. The system continuously records multiple physiological parameters associated with stress and emotional arousal, including:

- heart rate (HR);
- heart rate variability (HRV);
- electrodermal activity (EDA);
- skin temperature;
- movement and activity levels.

The biometric device operates independently from the VR system but is time-synchronized with the experimental software, ensuring precise temporal alignment between physiological signals and VR events. Both the VR application and the PC-based control software automatically generate detailed time-stamped log files, including:

- environment configuration parameters;
- audience size and room type;
- All AI-generated questions are delivered to the participant;
- session start and end times;
- user interaction events within the VR environment.

**Fig. 1:**
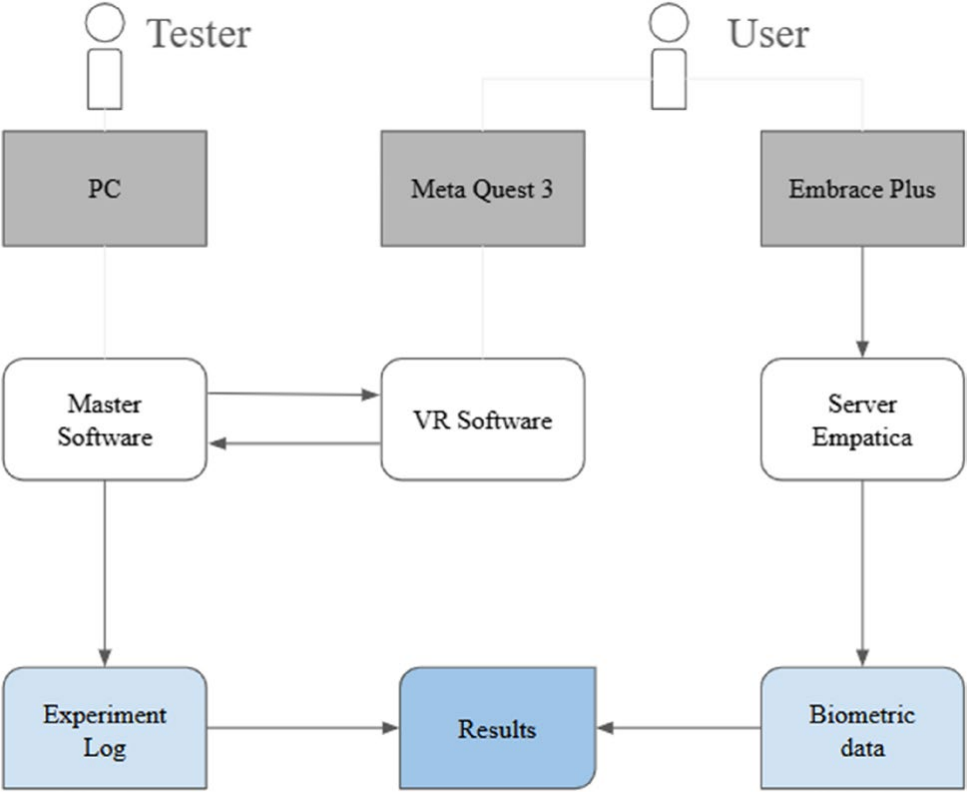
A visual representation of the computational workflow of the experiment

After each session, biometric data collected from the Empatica platform and behavioral and system-level data from the experimental software are exported and temporally aligned using synchronized timestamps. This enables precise mapping between physiological responses and specific experimental events (e.g., question onset, manipulation of audience size), allowing the identification of biometric markers associated with public speaking anxiety and stress responses. An overview of the system architecture is presented in Fig. 1.

### Protocol

**Fig. 2:**
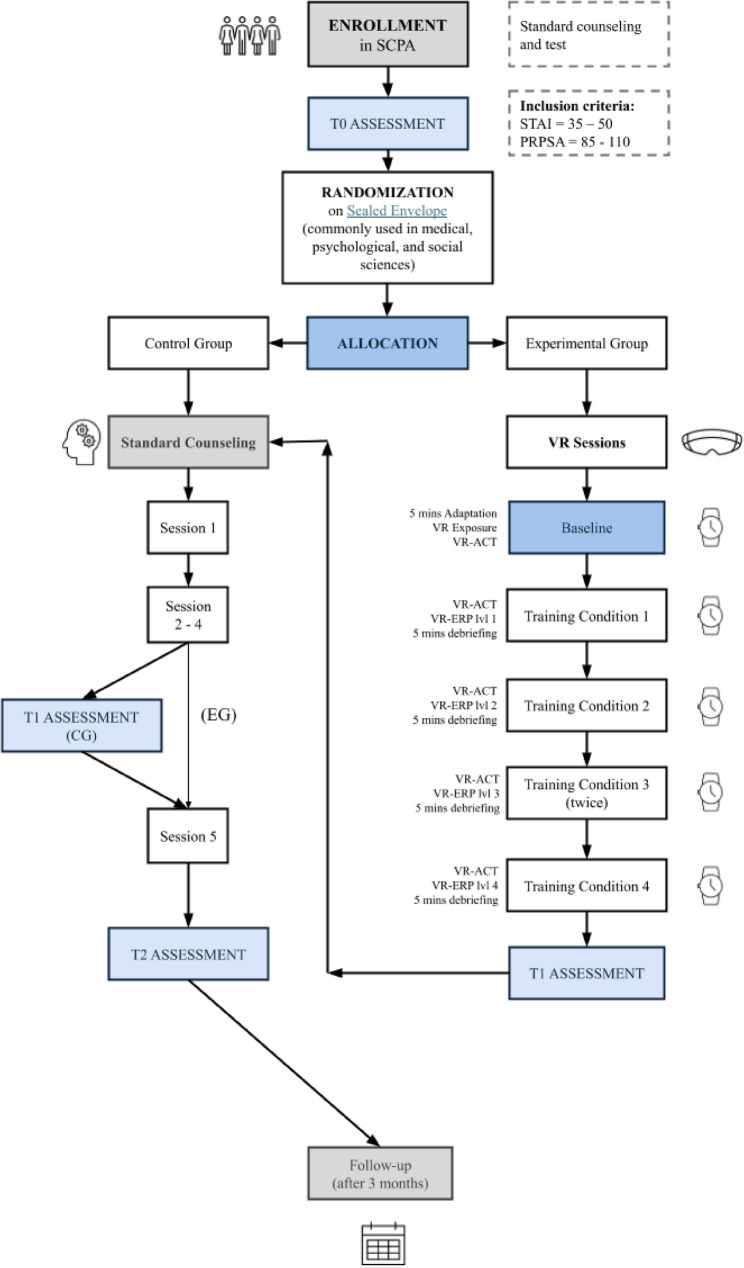
Workflow of the randomized controlled trial

The study adopts a randomized pretest–posttest control group design with two parallel conditions. Participants are university students accessing the UPCS who report experiencing anxiety and/or stress and meet the eligibility criteria. After a structured screening interview, eligible students provide written informed consent and are then allocated to either the experimental group or the control group through a randomization procedure (Fig. 2). Randomization was performed using Sealed Envelope software with variable block sizes, stratified by gender and site. The experimental group receives a standard psychological counseling program complemented by immersive VR modules, whereas the control group will receive the counseling program only. Participants complete the same set of standardized measures at pretest (before the intervention) and posttest (after the intervention) to allow for within-group and between-group comparisons over time (Fig. 2).

The study schedule and assessments are described in detail in Table 1 in accordance with the SPIRIT (Standard Protocol Items: Recommendations for Intervention Trials) guidelines. The completed SPIRIT checklist is provided as Supplementary Material (Table S1).

**Table 1:** SPIRIT schedule

Study period	Enrolment	Allocation	Week 1 (T0)	Week 2	Week 3	Week 4 (T1)	T2 (3-mo FU)
ENROLMENT							
Eligibility screen (structured interview)	✓						
Informed consent	✓						
Baseline demographics / clinical info	✓						
ALLOCATION							
Randomization (1:1)		✓					
INTERVENTIONS							
VR-ERP (experimental arm)			✓	✓	✓	✓	
VR-ACT (experimental arm)			✓	✓	✓	✓	
Control arm: counseling only			✓	✓	✓	✓	
ASSESSMENTS							
Demographics and academic data	✓						
PRPSA	✓					✓	✓
STAI-Y State	✓					✓	✓
STAI-Y Trait	✓					✓	✓
Physiological recording (EmbracePlus) • Heart rate (HR); • Heart rate variability (HRV); • Electrodermal activity (EDA); • Skin temperature; • Movement and activity levels.			✓	✓	✓	✓	
Adverse events / discomfort			✓	✓	✓	✓	
Withdrawal	✓	✓	✓	✓	✓	✓	✓

### Interventions

#### Participants

Participants in the control group do not receive VR training but follow the same assessment schedule as the experimental group. Specifically, they complete baseline (T0), post-intervention (T1), and follow-up (T2) evaluations using the same psychological measures. This design allows comparison between intervention and control conditions, thereby isolating the effects of VR training from potential confounding factors such as time and repeated testing effects.

The experimental intervention includes two distinct VR-based training components: (A) VR-ERP, which gradually exposes students to anxiety-provoking situations to prevent avoidance responses, and (B) VR-ACT training, which provides consistent mindfulness practice aimed at enhancing psychological flexibility and value-oriented behaviors. After completing VR-ACT training, participants in the experimental group proceed to a standard counseling intervention.

#### Control arm – counseling program

Standard counseling sessions are conducted by a psychotherapist for five consecutive weeks, consisting of 60-minute individual sessions. Sessions are structured as follows:

Session one: The therapist builds the therapeutic alliance and promotes acceptance and psychological flexibility processes within a non-judgmental and safe space. The session includes psychoeducation on the function of social anxiety and the distinction between avoidance and value-oriented behaviors.

Sessions two to four: The psychotherapist promotes self-as-context, cognitive defusion and restructuring, contact with the present moment, values clarification, acceptance, and committed action to transform the relationship with difficult emotions and to enhance behavioral activation and emotion regulation. Sessions include an individualized case formulation and between-session homework assignments.

Session five: The psychotherapist focuses on consolidation and relapse prevention and administers the post-intervention assessment (T1).

Follow-up session: Participants in both conditions complete follow-up assessments 3 months post-intervention to evaluate the stability of treatment effects and detect delayed or emerging improvements. These structured touchpoints also allow counselors to identify residual or emerging difficulties, thereby strengthening ecological validity and clinical utility.

#### Experimental arm – VR-based training scenarios

VR is employed for a dual therapeutic purpose:

1. simulating feared, anxiety-evoking scenarios, thereby enabling both assessment and training of situationally induced anxiety and progressively promoting desensitization to anxiety-provoking stimuli;
2. delivering an ACT-consistent mindfulness training targeting psychological flexibility processes (e.g., contact with the present moment, cognitive defusion, and acceptance), eliciting a grounding response within a digitally generated and safe environment.

**Fig. 3:**
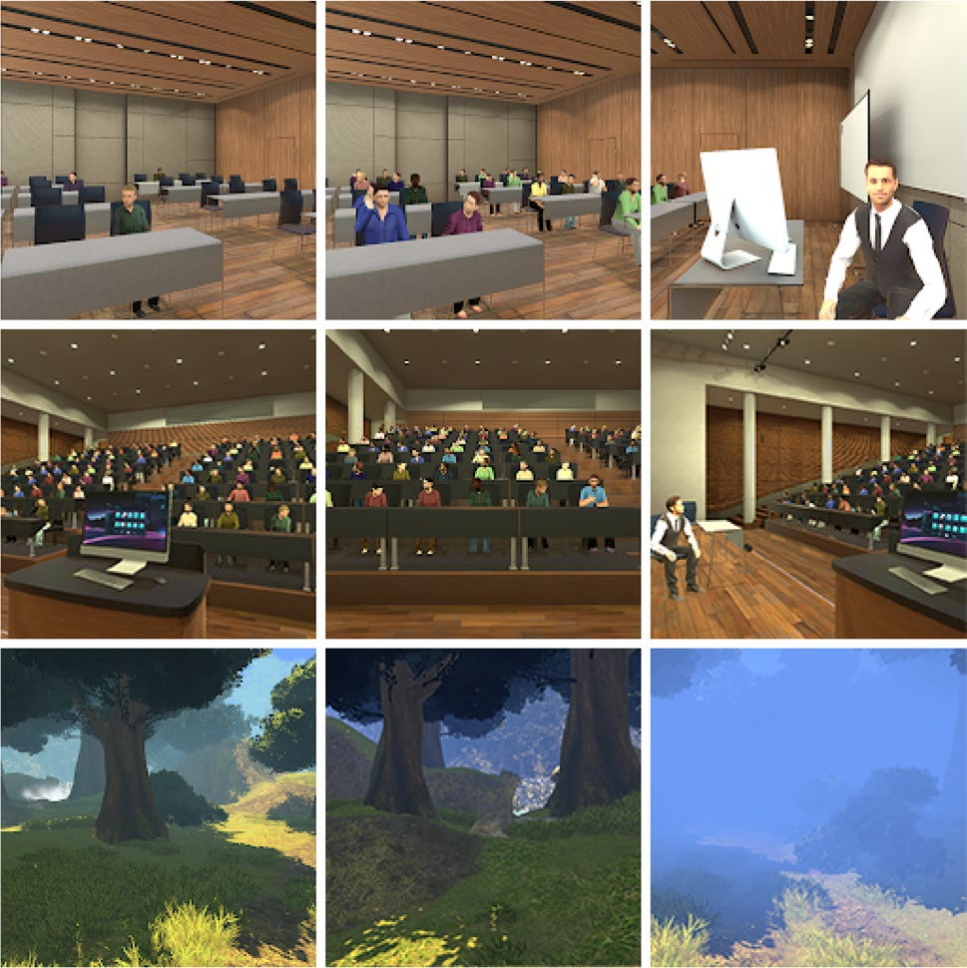
Representative first-person views from the VR application. Top row: classroom scene with low audience density, full audience, and view of the professor. Middle row: user view from the lectern, front view of the lecture hall audience, position of the professor relative to user. Bottom row: forest relaxation environment showing progressive fog proximity used for respiration-synchronized mindfulness guidance

VR sessions are divided into three sequential phases (total duration approximately 15 min): Adaptation, Exposure, and Relaxation (Fig. 3).

Adaptation phase (5 min): Participants are guided through brief relaxation and grounding exercises and are familiarized with the VR equipment. Procedures for operating the devices (described in subsequent sections) are explained, and participants are encouraged to become comfortable in the immersive environment to facilitate engagement and reduce novelty-related discomfort during subsequent phases.

Exposure phase (5 min): This phase constitutes the core component of the VR-based intervention. Participants are exposed to simulated public speaking situations embedded within an examination context. Across sessions, task difficulty and social-evaluative stress are gradually increased through systematic manipulation of virtual characters (e.g., number and behavior of avatar instructors and audience members). This graded exposure supports progressive acquisition and practice of skills to manage anxiety and stress related to examinations.

Relaxation phase (5 min): This phase is dedicated to mindfulness practice and rebalancing, aiming to reduce stress accumulated during exposure. Mindfulness practice supports inner awareness and improves emotion and stress management. The relaxation component also facilitates consolidation of the intervention’s effects on anxiety and stress regulation.

#### VR-ACT training

The VR-ACT experience takes place in a 360° immersive 3D naturalistic woodland environment (Fig. 3c), surrounded by trees and natural elements designed to evoke calm and grounding. Dynamic interactive features—such as breath-synchronized fog, ambient nature-based auditory stimuli, and subtle continuous movements of light, leaves, and grass—enhance engagement and perceived presence during a semi-structured mindfulness training session. The session targets ACT psychological flexibility processes and fosters feedback-based coordination between participants and the virtual setting, promoting self-regulation and adaptive learning of psychological responses aligned with each targeted mindfulness skill. Phase duration is determined by the operator, allowing exploration of different options within each phase and enhancing flexibility of the immersive experience.

#### VR-ERP

VR-ERP uses 360° 3D videos depicting scenarios such as an exam classroom and an auditorium within a university campus (Fig. 3a–b). Across sessions, variables such as audience size and behavior, professor characteristics, attire (formal vs. informal), and examination committee composition vary. Scenarios are presented according to a gradual hierarchy of challenge; at the student’s discretion, specific scenarios are repeated. Scenarios are selected based on distressing contents identified in the research-intervention literature [29]. During simulations, participant interactions influence the subsequent course of the exposure scenario. Each scenario follows a graded increase in exposure intensity, culminating in an oral exam involving a PowerPoint presentation in the final exposure. Each VR-ERP session is preceded by a VR-ACT session and followed by a 10-minute debriefing intervention providing supportive feedback and guidance to consolidate skills acquired during exposure.

Familiarization session: in the session participants receive a standardized explanation of the VR program’s aims and procedures. They are introduced to the Oculus Quest 3, a consumer-grade standalone VR system consisting of a head-mounted display (HMD) and two handheld controllers. After donning the headset, they complete a brief 5-minute familiarization session to learn basic controls and navigation. This training supports acclimatization to the VR environment, facilitates graded exposure to performance-related stressors, and promotes adaptive regulation of physiological and affective responses within a controlled yet ecologically valid simulation.

Baseline session: the session establishes a physiological reference point preceding implementation of the training. The configuration elicits a controlled anxiogenic response associated with judgment, self-presentation, and evaluation expectancy. Physiological measures provide a baseline stress reactivity index under standardized conditions and support subsequent assessment of training-related modulation of autonomic responses.

Training Condition 1: The condition introduces the exam-related context in a minimally threatening and highly controlled manner. The configuration presents the student and professor within an examination room (Fig. 3, top row, first column), while the interaction remains informal and limited to non-evaluative questions. The condition elicits mild activation of evaluative schemas, allowing engagement with oral exam cues without triggering a marked anxiogenic response. The focus remains on encouraging adaptive self-regulation and facilitating habituation to interpersonal and environmental components of the scenario. By reducing perceived pressure and discouraging avoidance or neutralizing strategies, this phase provides a foundational step toward more complex and emotionally salient training conditions.

Training Condition 2: The condition intensifies exposure to social-evaluative cues within the examination context. The scenario occurs in an exam room and includes the student, the professor, and an audience of approximately ten observers (Fig. 3, top row, middle column). Attire remains informal, but the interaction shifts to actual exam-related questions, increasing perceived performance demands. This configuration amplifies engagement with social evaluation and performance pressure while preventing neutralization and covert avoidance behaviors. Sustained exposure under controlled but more challenging conditions supports physiological habituation and consolidation of adaptive behavioral self-regulation, preparing participants for higher-intensity exposures in subsequent phases.

Training Condition 3: The condition maximizes exposure to high-intensity social-evaluative demands within a formal examination setting. The session takes place in a large lecture hall and involves the student, professor, and a broader audience (Fig. 3, middle column, middle row). All characters wear formal attire, and the interaction centers on actual exam questions, increasing realism and performance salience. This configuration facilitates extinction of phobic responses and promotes development of adaptive coping strategies within a highly interactive, evaluatively charged environment. The condition encourages expansion of the behavioral repertoire and implementation of active self-regulation strategies. As the most demanding phase of the protocol, it supports consolidation of robust and context-generalizable regulation skills.

Training Condition 4: The condition promotes generalization and consolidation of responses acquired in preceding phases and tests whether self-regulation skills, coping strategies, and reduced anxiety responses extend to a related but distinct high-salience social performance context. The condition takes place in a large lecture hall and is framed by a formal examination atmosphere. The scenario includes the student, professor, examination committee, and an audience. The task shifts from answering exam questions to delivering a presentation, introducing a different performance format. This configuration requires autonomous organization, sustained delivery, and direct engagement with an evaluative audience, providing a stringent test of stability and transferability of learned regulatory processes.

### Statistical analyses

All statistical analyses are conducted using IBM SPSS Statistics (version 29.0) and follow a pre-specified analytic strategy designed to evaluate changes in psychological and physiological outcomes across the study period and to compare the effectiveness of standard counseling with and without VR-based exposure. Descriptive statistics are first computed to characterize the sample to assess baseline equivalence between groups. Reliability analyses (Cronbach’s α and McDonald’s ω) are performed to confirm the internal consistency of the psychometric instruments.

Because the study involves repeated assessments at baseline (T0), post-intervention (T1), and follow-up (T2), the primary analyses use linear mixed-effects models, an approach well suited to handling longitudinal data and missing observations. These models test for the main effects of time and group, as well as the critical time × group interaction, indicating differential improvement between the VR-enhanced counseling condition and the control group. Where significant interactions emerge, post hoc comparisons with Bonferroni adjustment are conducted to identify specific temporal patterns of change. Complementary analyses of change scores (ΔT1–T0 and ΔT2–T0) are performed using independent-sample t-tests or, where assumptions are violated, Mann–Whitney U tests; General Linear Models including baseline variables and demographic covariates further clarify group differences in treatment response.

Physiological data collected during VR sessions (including heart rate, HRV, and electrodermal activity) are examined through mixed models and event-based analyses aligned with the structure of the VR exposure tasks, allowing evaluation of both arousal patterns and recovery processes. Exploratory regression models are used to identify predictors of treatment benefit, such as baseline symptom severity or physiological reactivity during VR exposure.

Missing data are handled through the mixed-model framework, which accommodates incomplete longitudinal data; if dropout shows non-random patterns, multiple imputation is applied as a sensitivity check. For all analyses, statistical significance is set at α = 0.05, with appropriate corrections for multiple comparisons.

## Discussion

Public speaking anxiety is one of the most prevalent forms of anxiety among students in higher education [30]. Overall, research across countries indicates that public speaking and presentation tasks are among the most anxiety-provoking learning situations in higher education. High proportions of students report significant fear or anxiety in these contexts, and even preliminary evidence suggests that, for some, symptoms can reach clinically meaningful levels [31, 32].

This project attempts to provide university students accessing the UPCS with a safe, controlled, and ecologically valid environment to practice managing PSA in realistic but non-threatening academic contexts, such as exams, research presentations, and oral defenses. Grounded in emotional processing theory [33] and the cognitive-behavioral framework of exposure therapy [34], the intervention leverages a VR-based treatment to elicit anxiety-related responses within a controllable context. This approach allows participants to gradually face and regulate stress while developing adaptive coping mechanisms related to public speaking anxiety. From a scientific standpoint, the project contributes to growing evidence that VR-based interventions can effectively simulate socially evaluative situations, enhancing self-efficacy, emotional regulation, and desensitization processes in individuals with PSA and related forms of performance anxiety [35, 36]. Specifically, the project aims to highlight how the combination of standard counseling with VR-based ERP and ACT-consistent training may enhance therapeutic effectiveness beyond what either approach could achieve alone. By embedding VR-based exposure within an ongoing counseling relationship, the intervention allows feared public speaking situations to be addressed in a graded, controlled, and ecologically valid manner, while simultaneously promoting reflective processing, emotional meaning-making, and values-based through therapist support.

The hypotheses of the study are grounded in the assumption that VR-based interventions and standard counseling exert their effects through partially distinct but complementary mechanisms. Specifically, the VR-ERP components are expected to primarily engage inhibitory learning and habituation processes, facilitating repeated confrontation with feared public speaking situations while preventing avoidance behaviors [37]. This mechanism is hypothesized to result in a reduction of fear, threat expectancy, and perceived cognitive load during performance-related tasks. In parallel, the VR-ACT components, particularly the grounding and mindfulness-based modules, are expected to predominantly influence physiological arousal and somatic tension, fostering greater emotional regulation, acceptance of internal experiences, and present-moment awareness during exposure [38]. The integration of these processes within standard counseling is expected to promote both immediate symptom reduction and durable psychological flexibility, thereby accounting for the persistence of treatment effects observed at follow-up. Together, these findings would support a process-based model in which embodied regulation and experiential exposure jointly contribute to sustained improvements in public speaking anxiety and stress-related outcomes.

The use of real-life–like VR scenarios enables repeated exposure to socially evaluative contexts that closely approximate everyday academic demands, such as oral presentations and audience scrutiny, thereby strengthening generalization to real-world functioning [39, 40]. Moreover, the integration of ACT-based VR exercises provides a complementary mechanism by fostering acceptance of internal experiences, cognitive defusion, and present-moment awareness during exposure, rather than focusing exclusively on symptom reduction [41]. From a technological perspective, the project fosters innovation in human–machine interaction and the use of real-time biofeedback to support mindfulness and stress regulation. Future developments could incorporate artificial intelligence–driven adaptive content to personalize exposure intensity and virtual instructor feedback, increasing ecological validity and therapeutic responsiveness. The integration of quantitative (psychophysiological and psychometric) and qualitative (subjective experience) data will provide a comprehensive understanding of VR’s impact on anxiety management and user engagement.

This study has the potential to help address factors associated with university dropout rates linked to anxiety, contribute to improvements in overall psychological well-being among university students, and offer an accessible, and potentially cost-effective alternative to traditional psychotherapy. Additionally, the project strengthens interdisciplinary collaboration between psychology, engineering, and computer science students, fostering professional competencies in digital mental health design.

Some potential limitations and practical challenges may arise during the implementation of this study. One limitation of this study is its requirement for high participant commitment, which may have reduced engagement and increased attrition, thereby limiting the generalizability of the findings. Another limitation concerns the sample composition, as the study focuses on help-seeking university students, a group that may differ in motivation, access to technology, and psychological awareness compared to the general population or younger adolescents. This restricts the ability to generalize the results to other demographic or clinical groups. Finally, the relatively short follow-up period restricts conclusions regarding the long-term maintenance and generalization of treatment gains to real-world academic performance contexts.

Despite these constraints, the study is expected to provide valuable preliminary evidence regarding the feasibility, acceptability, and potential efficacy of VR-based interventions for anxiety management within higher education. The results will help inform the design of larger randomized controlled trials and support the development of scalable, evidence-based digital interventions that integrate VR with established therapeutic approaches, with the aim of enhancing accessibility, engagement, and personalization in mental health care.

## Supplementary Information

Supplementary Material 1.

## Data Availability

No datasets were generated or analysed during the current study.

## Abbreviations

ACT: Acceptance and Commitment Therapy
BVP: Blood Volume Pulse
CBT: Cognitive Behavioral Therapy
EDA: Electrodermal Activity
ERP: Exposure and Response Prevention
HMD: Head-mounted Display
HR: Heart Rate
HRV: Heart Rate Variability
IBI: Inter-Beat Interval
IVR: Immersive Virtual Reality
PPG: Photoplethysmography
PRPSA: Personal Report of Public Speaking Anxiety
PSA: Public Speaking Anxiety
RCT: Randomized Controlled Trial
STAI: State-Trait Anxiety Inventory
UPCS: University Psychological Counseling Service
VR: Virtual Reality
VRET: Virtual Reality Exposure Therapy
